# ASL expression in ALDH1A1^+^ neurons in the substantia nigra metabolically contributes to neurodegenerative phenotype

**DOI:** 10.1007/s00439-021-02345-5

**Published:** 2021-08-21

**Authors:** Shaul Lerner, Raya Eilam, Lital Adler, Julien Baruteau, Topaz Kreiser, Michael Tsoory, Alexander Brandis, Tevie Mehlman, Mina Ryten, Juan A. Botia, Sonia Garcia Ruiz, Alejandro Cisterna Garcia, Carlo Dionisi-Vici, Giusy Ranucci, Marco Spada, Ram Mazkereth, Robert McCarter, Rima Izem, Thomas J. Balmat, Rachel Richesson, Members of the UCDC, Matthias R. Baumgartner, Jirair K. Bedoyan, Gerard Berry, Susan A. Berry, Peter Burgard, Lindsay Burrage, Curtis Coughlin, George A. Diaz, Gregory Enns, Renata C. Gallagher, Andrea Gropman, Cary O. Harding, Georg Hoffmann, Cynthia Le Mons, Shawn E. McCandless, J. Lawrence Merritt, Sandesh C. S. Nagamani, Andreas Schulze, Jennifer Seminara, Tamar Stricker, Mendel Tuchman, Susan Waisbren, James D. Weisfeld-Adams, Derek Wong, Marc Yudkoff, Ehud Gazit, Sandesh C. S. Nagamani, Ayelet Erez

**Affiliations:** 1 Department of Biological Regulation, Weizmann Institute of Science, Rehovot, Israel; 2 Department of Veterinary Resources, Weizmann Institute of Science, Rehovot, Israel; 3 Great Ormond Street Institute of Child Health, and NIHR Great Ormond Street Hospital Biomedical Research Centre, University College London, London, UK; 4 Great Ormond Street Hospital for Children, NHS Foundation Trust, London, UK; 5 The Shmunis School of Biomedicine and Cancer Research, George S. Wise Faculty of Life Sciences, Tel Aviv University, 6997801 Tel Aviv, Israel; 6 Life Science Core Facility, Weizmann Institute of Science, Rehovot, Israel; 7 Institute of Neurology, University College London (UCL), London, UK; 8 Department of Information and Communications Engineering, University of Murcia, Murcia, Spain; 9 Division of Metabolism, Bambino Gesù Children’s Hospital IRCCS, Rome, Italy; 10 The Sackler School of Medicine, Tel-Aviv University, Tel-Aviv, Israel; 11 Center for Translational Sciences, Children’s National Health System, The George Washington University, Washington, D.C., USA; 12 Children’s National Medical Center, Washington, D.C., USA; 13 Research Computing, Duke University, Durham, NC USA; 14 Learning Health Sciences, University of Michigan, Ann Arbor, MI USA; 15 Department of Molecular and Human Genetics, Baylor College of Medicine, Houston, TX USA; 16 Texas Children’s Hospital, Houston, TX USA

## Abstract

Argininosuccinate lyase (ASL) is essential for the NO-dependent regulation of tyrosine hydroxylase (TH) and thus for catecholamine production. Using a conditional mouse model with loss of ASL in catecholamine neurons, we demonstrate that ASL is expressed in dopaminergic neurons in the substantia nigra pars compacta, including the ALDH1A1^ +^ subpopulation that is pivotal for the pathogenesis of Parkinson disease (PD). Neuronal loss of ASL results in catecholamine deficiency, in accumulation and formation of tyrosine aggregates, in elevation of α-synuclein, and phenotypically in motor and cognitive deficits. NO supplementation rescues the formation of aggregates as well as the motor deficiencies. Our data point to a potential metabolic link between accumulations of tyrosine and seeding of pathological aggregates in neurons as initiators for the pathological processes involved in neurodegeneration. Hence, interventions in tyrosine metabolism via regulation of NO levels may be therapeutic beneficial for the treatment of catecholamine-related neurodegenerative disorders.

**Supplementary Information:**

The online version contains supplementary material available at 10.1007/s00439-021-02345-5.

## Introduction

In the liver, the urea cycle enzymes argininosuccinate lyase (ASL) and argininosuccinate synthase 1 (ASS1) are required to convert waste-nitrogen to urea. Loss of ASL or ASS1 activity causes argininosuccinic aciduria (or argininosuccinate lyase deficiency, ASLD, MIM#207900) and citrullinemia type 1 (MIM#215700), respectively. These two disorders are a subset of the classical inborn errors of metabolism called urea cycle disorders (UCD), characterized by episodes of hyperammonemia (1–4). In tissues other than the liver, ASL and ASS1 participate in the arginine-citrulline cycle, which generates arginine, an essential metabolic nexus for synthesizing critical metabolites such as creatine, proline, polyamines, glutamate, and nitric oxide (NO). While ASL and ASS1 participate in the arginine-citrulline cycle, ASL is the only enzyme in mammals that can endogenously synthesize arginine (5, 6).

Individuals with germline pathogenic variants in *ASL* can have neurocognitive deficits, seizures, and motor abnormalities (7). Whereas hyperammonemia is a significant determinant for overall neurological outcomes in ASLD, some neurocognitive deficits and behavioral abnormalities have also been reported in individuals without substantial hyperammonemia, suggesting that they may result from a neuronal tissue-autonomous loss of ASL. We have recently reported that ASL deficiency in the locus coeruleus (LC) results in impaired NO synthesis, decreased tyrosine hydroxylase (TH) activity, and consequently, in low dopamine and norepinephrine levels (8). Notably, both dopamine and norepinephrine are important neurotransmitters (9–11), and their deficiency has been associated with neurodegenerative disorders, including Parkinson's Disease (PD) (12, 13).

LC projects to the ventral tegmental area (VTA) and the substantia nigra pars compacta (SNc). These projections are highly interconnected and are necessary for the survival of vulnerable midbrain dopaminergic neurons (14–16). Loss of these projections has been shown to facilitate neuronal degeneration and is responsible for some of the motor and non-motor symptoms associated with PD (12–14, 17). Specifically, within the SNc, a subpopulation of dopaminergic neurons express aldehyde dehydrogenase, i.e., ALDH1A1^**+**^-neurons. These neurons require ALDH1A1 for their normal functioning and survival (18–20); their preferential degeneration has been shown to contribute to the pathogenesis of PD and has been implicated explicitly in movement impairments associated with PD (21, 22). Furthermore, ALDH1A1 levels in peripheral blood have been used as a biomarker for PD (23).

PD is characterized by impaired catecholamine levels and the formation of amyloidogenic proteins and α-synuclein aggregates in the SNc (24, 25). Interestingly, the fundamental processes that are involved in the initiation and propagation of these protein aggregates remain elusive (26–28); it has been suggested that the accumulation of small metabolites acts as "seeds" for the formation of the aggregates (29). Based on these data from the literature, we hypothesized that decreased activity of ASL and TH would contribute to the pathogenesis of neurodegeneration by perturbing tyrosine metabolism.

## Results

### ASL is distinctively expressed in TH—ALDH1A1+- SNc neurons

**Fig. 1:**
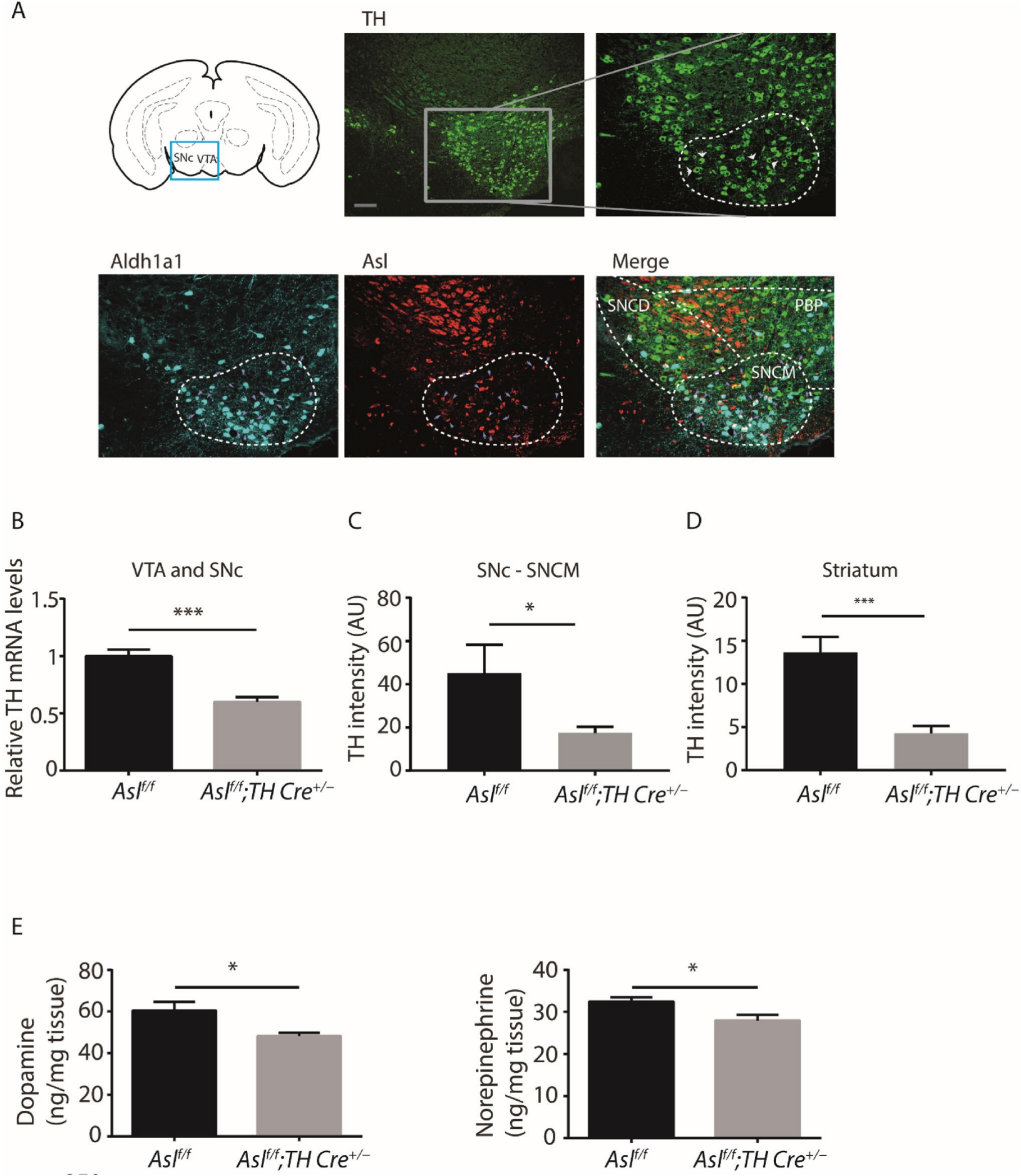
ASL co-localizes with TH and ALDH1A1 in the SNc, and its deficiency is associated with decreased TH expression and catecholamine synthesis. **A** VTA and SNcM dopaminergic regions are demonstrated in a scheme of coronal midbrain section (bregma -3.65 mm) (top panel left) and by TH staining (top panel middle, green) (Scale bar = 500 µm). Magnification of the box area indicates TH (top panel right, green), ALDH1A1 (lower panel left, cyan), ASL (lower panel middle, red), and their co-staining (lower panel right). Dashed lines differentiate the SNcM and VTA subregions (Scale bar = 250 µm). Arrows point to representative cells co-expressing the three proteins. **B** Quantification of *TH* mRNA in the SNcM and VTA regions of *Asl*^*f/f*^*; TH Cre*^+*/−*^ and in *Asl*^*f/f*^ control mice as measured by RT-PCR with specific TaqMan probes (*n* = 7 mice in each group). **C**, **D** Quantification of TH immunostaining of TH- ALDH1A1^**+**^ neurons in the SNc medial (SNcM) (**C**) and striatum (**D**) of *Asl*^*f/f*^*; TH Cre*^+*/−*^ and *Asl*^*f/f*^ control mice (*n* = 5). **E** Measurement of SNcM catecholamine levels shows a reduction in both dopamine (left) and norepinephrine (right) in *Asl*^*f/f*^*; TH Cre*^+*/−*^ as compared to *Asl*^*f/f*^ control mice (*n* ≥ 9 mice in each group). SNCD-SNc dorsal, PBP-parabrachial pigmented nucleus. Data represent mean ± s.e.m. (*p < 0.05, **p < 0.01, ***p < 0.001, ****p < 0.0001; *ns* not significant)

By immunostaining in dopaminergic neurons in wild-type mice, we found a distinctive pattern of expression for ASL in TH -ALDH1A1^**+**^- SNc neurons (Fig. 1A and Supp. Figure 1A). Conditional ASL knockout mice, i.e., *Asl*^*f/f*^*; TH Cre*^+*/−,*^ did not express ASL in this unique TH ALDH1A1^+^sub-population of neurons (Supp. Figure 1A). Interestingly, these SNc-ALDH1A1^+^neurons have been shown to undergo degeneration in PD and have been linked directly with the motor impairments associated with PD (18, 30). Using laser microdissection, we collected TH positive neurons from the SNc and VTA (Supp. Figure 1B). Corroborating our previous findings (8), following *Asl* deletion (Supp. Figure 1C), we found a reduction in *TH* mRNA levels in *Asl*^*f/f*^; *TH Cre*^+*/−*^ mice as compared to control mice (*Asl*^*f/f*^) (Fig. 1B). Immunostaining of SNc- ALDH1A1^+^neuronal sub-population demonstrated a reduction in TH protein levels, specifically in the SNc of *Asl*^*f/f*^; *TH Cre*^+*/−*^ compared to control *Asl*^*f/f*^ mice (Fig. 1C and Supp. Figure 2A). As SNc- ALDH1A1^+^neurons are known to project predominantly to the dorsolateral striatum (22, 31), we evaluated the expression of TH in ALDH1A1^+^ fibers in this region; we found that *Asl*^*f/f*^; *TH Cre*^+*/−*^ mice show a significant reduction in TH levels in the dorsolateral striatum as well, in comparison to control mice (Fig. 1D). To test whether the downregulation of TH in dopaminergic neurons is functionally significant, we measured catecholamine levels in punch biopsies taken from the SNc region and found that *Asl*^*f/f*^; *TH Cre*^+*/−*^ have lower levels of dopamine and norepinephrine in comparison to *Asl*^*f/f*^ control mice (Fig. 1E). Additionally, cerebrospinal fluid (CSF) analysis from adult *Asl*^*f/f*^; * TH Cre*^+*/−*^ mice revealed elevated levels of ALDH1A1 protein in comparison to control mice (Supp. Figure 2B). Altogether, Asl plays an essential role in synthesizing and secretion of dopamine from SNc-ALDH1A1^+^ neurons to the dorsolateral striatum.

### ASL deficiency is associated with motor deficits in mice with loss of ASL in TH—ALDH1A1^+^- SNc neurons and in individuals with ASLD

**Fig. 2:**
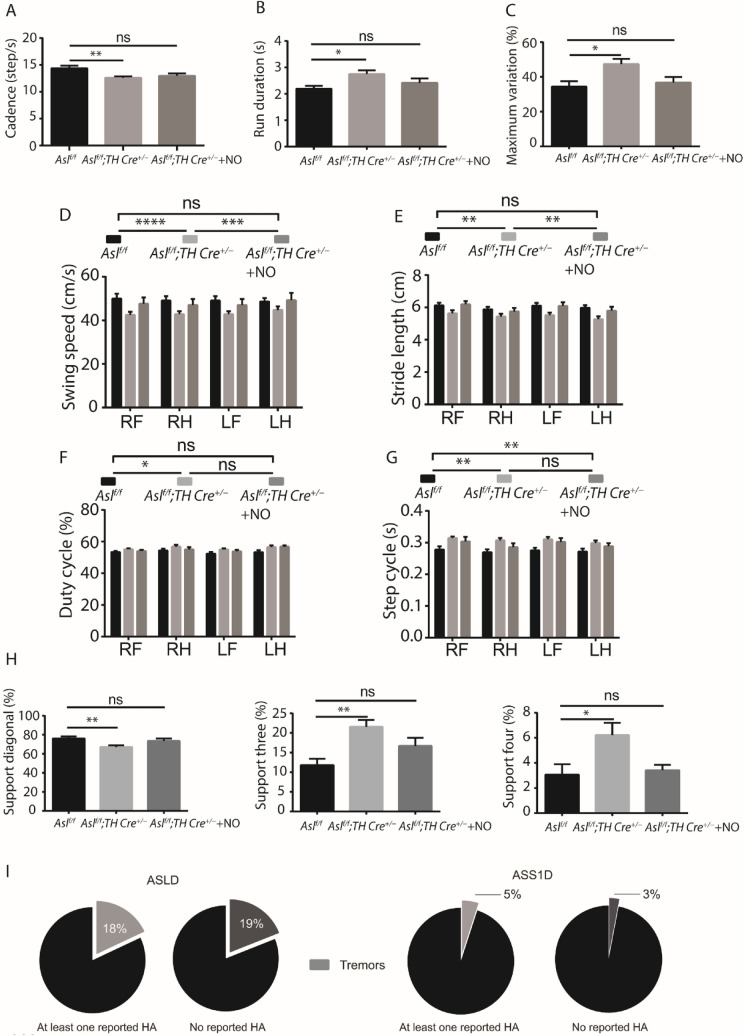
ASL deficiency results in motor dysfunctions in both humans and rodents. **A**–**H** Gait analysis in *Asl*^*f/f*^*;TH Cre*^+*/−*^ and *Asl*^*f/f*^ control mice. Dynamic paw parameters included: **A** Cadence. **B** Run duration. **C** Maximum variation. **D** Swing speed. **E** Stride length **F** Duty cycle. **G** Step cycle. **H** Paws support. (*n* $\ge$ 10). **I** Pie charts demonstrating the percentage of subjects with tremors in ASLD (left part) and ASS1D (right part) patients with or without documented episode of HA. Data represent mean ± s.e.m. (*p < 0.05, **p < 0.01, ***p < 0.001, ****p < 0.0001; *ns* not significant)

The decreased catecholamine levels in the SNc region led us to examine motor functions in *Asl*^*f/f*^*; TH Cre*^+*/−*^ mice. Using gait pattern assessment test ("CatWalk™ Gait Analysis" (32)), we found significant abnormalities in *Asl*^*f/f*^*; TH Cre*^+*/−*^ mice in ten different gait parameters that are commonly found in rodent models of PD (33) (32) (Fig. 2A–H). Nevertheless, as ASL is expressed in other catecholamine neurons, the gait deficits may be caused by a synergistic effect between the ablation of ASL from both ALDH1A1^+^ and other additional dopaminergic neurons. Multiple studies by us and others have shown that ASL is essential for NO generation and that ASL loss leads to NO-related pathologies, including cognitive deficits (5, 7, 8, 34, 35). Specifically, in catecholamine neurons, dysregulation of NO signaling has been shown to alter dopamine production (8) (36). Hence, we next aimed to rescue the phenotypic consequences of ASL loss from dopaminergic neurons by supplementing *Asl*^*f/f*^*; TH Cre*^+*/−*^ mice with NOS-independent NO donors. Encouragingly, adding NO supplement was sufficient to rescue many abnormal gait parameters (Fig. 2A–H).

To evaluate whether our findings are relevant to humans with ASLD, we mined the clinical data collected by the NIH Rare Disease Clinical Research Network's Urea Cycle Disorders Consortium. We identified that a higher proportion of individuals with ASLD (23 out of 124, 18%) are reported to have tremors, as compared to individuals with ASS1D (5 out of 106, 5%) (*p* < 0.05) (Fig. 2I). Since hyperammonemia (HA) can confound many of the behavioral, cognitive, and motor outcomes in UCDs, we further evaluated the prevalence of tremors in these two subsets of UCDs in individuals who did not have any documented episodes of HA. The percentage of individuals with ASLD and ASS1D without at least one documented episode of HA was 42 (*n* = 52) and 28 (*n* = 29), respectively. In the absence of documented HA, the percentage of individuals with ASLD who had tremors was 19% (10 out of 52) as compared to 3% (1 out of 29) in ASS1D (Fig. 2I). Thus, the proportion of individuals with ASLD who reported tremors was higher than ASS1D, irrespective of history of presence or absence of documented HA.

### Loss of ASL in TH—ALDH1A1^+^- SNc neurons results in memory deficits

**Fig. 3:**
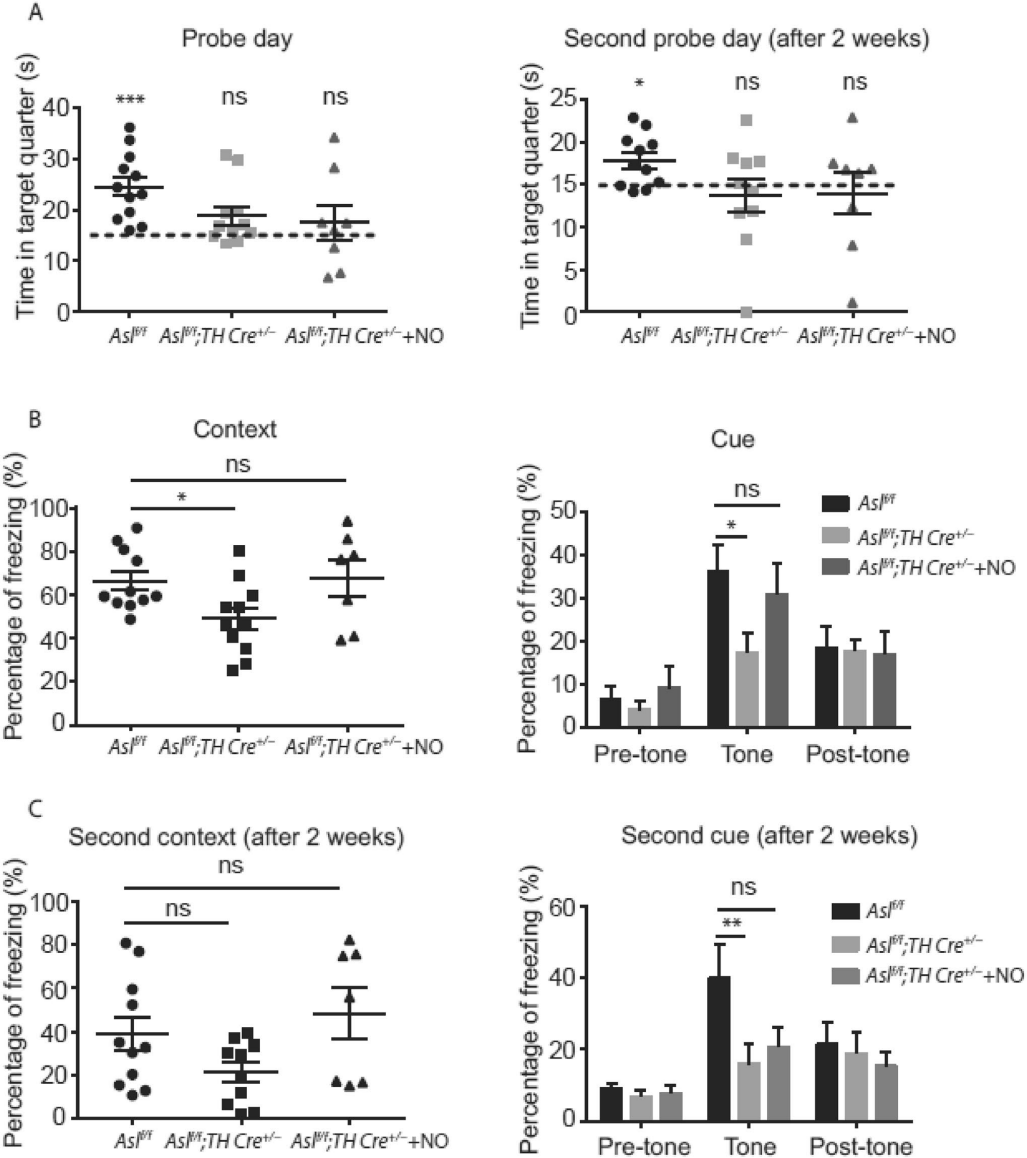
Adult *Asl*^*f/f*^*; TH Cre*^+*/−*^ mice display cognitive deficits that can be partially rescued with NO donors. **A** Adult *Asl*^*f/f*^ control group exhibit strong preference to the target quarter on probe day (left panel) and two weeks later (right panel), in comparison to adult *Asl*^*f/f*^*; TH Cre*^+*/−*^ mice. Acute treatment with NO donor did not rescue the performance of *Asl*^*f/f*^*; TH Cre*^+*/−*^ mice. The dashed line indicates the time expected by chance. **B** Adult control mice trained for the fear conditioning paradigm and tested for demonstrating freezing responses to context (left panel) and cue (tone) challenges (right panel), froze significantly more than adult *Asl*^*f/f*^*;TH Cre*^+*/−*^ mice. Acute treatment with NO donor rescues the performance of *Asl*^*f/f*^*;TH Cre*^+*/−*^ mice. **C** Two weeks following the first fear-conditioning assessment, mice were tested again and showed the same trend in both the context (left panel) and cue (right panel) tests. (n $\ge 8$). Data represent mean ± s.e.m. (*p < 0.05, **p < 0.01, ***p < 0.001, ****p < 0.0001; *ns* not significant)

To evaluate the memory of *Asl*^*f/f*^*; TH Cre*^+*/−*^ mice, we used the Morris water maze (MWM) and the fear conditioning test (37). In adult mice using the MWM test, we found that *Asl*^*f/f*^ control mice spent significantly more time in the target quarter as compared to *Asl*^*f/f*^*; TH Cre*^+*/−*^, while in younger mice, we did not observe any differences between the two genotypes (Fig. 3A, Supp. Figure 3A and data not shown). Notably, across the 7 days of learning, all mice showed similar learning behavior, suggesting that although adult *Asl*^*f/f*^*; TH Cre*^+*/−*^ are capable of learning, they have a problem in long-term memory consolidation (Supp. Figure 3B). We challenged the mice for a second time two weeks after the original probe day to confirm these findings. Here again, adult *Asl*^*f/f*^*; TH Cre*^+*/−*^ mice did not prefer searching the platform in the target quarter, while the *Asl*^*f/f*^ control mice recalled the platform location (Fig. 3A). In corroboration, when we challenged adult *Asl*^*f/f*^*; TH Cre*^+*/−*^ mice with the fear-conditioning paradigm for evaluating associative learning and memory, *Asl*^*f/f*^ control mice froze significantly more as compared to *Asl*^*f/f*^*; TH Cre*^+*/−*^ mice in both context and at the tone stage of the cue tests (Fig. 3B). Notably, the pre and post-tone stages did not show any difference, a trend that remained even 2 weeks after the initial testing (Fig. 3C). Together, these results imply that KO of ASL in catecholamine neurons of mice decreases memory-dependent performance.

To try and rescue these phenotypes, we supplemented the mice with NO donors for 2 weeks before the beginning of the experiments. Here, NO treatment significantly rescued *Asl*^*f/f*^*; TH Cre*^+*/−*^ mice only in the fear-conditioning test and less so in the MWM test (Fig. 3A–C).

### Decreased TH levels following ASL loss promotes tyrosine aggregates and increased α-synuclein levels

**Fig. 4:**
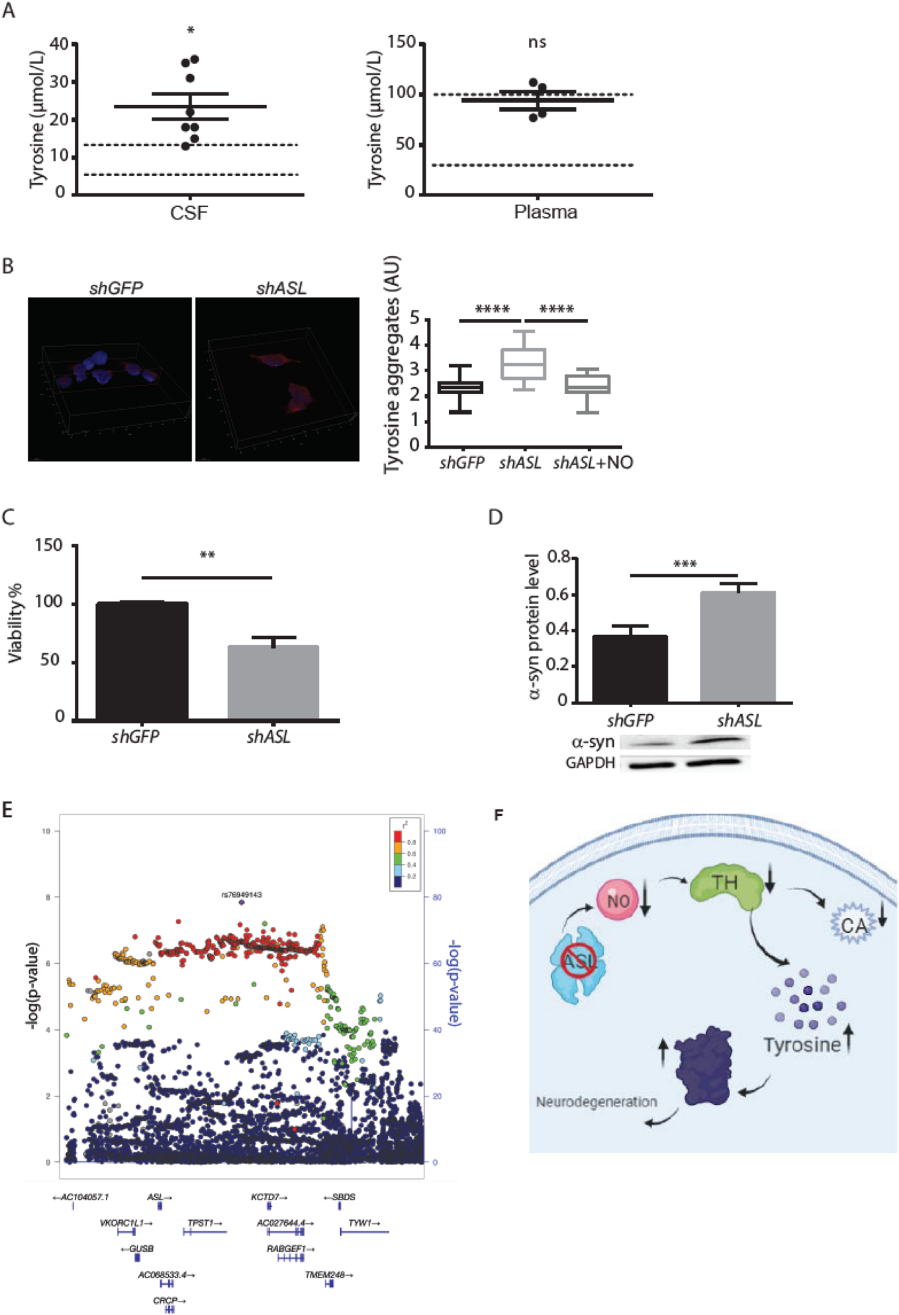
ASL deficiency in human patients associates with tyrosine accumulation and α-synuclein aggregation. **A** Tyrosine levels in the CSF (left panel) and plasma (right panel) of ASLD patients underwent liver transplantation. Dashed lines indicate the normal level range (*n* = 8 for CSF, *n* = 4 for plasma) (*p* < 0.02 for CSF, *p* = 0.56 for plasma). **B** SH-SY5Y neurons were stained with anti-tyrosine aggregates antibodies and visualized using confocal microscopy. Representative staining of DAPI (blue) and anti-tyrosine staining (red) are shown (Scale bars = 10 µm). A representative 3D volume reconstruction of the Z-series with XZ-slice projection staining of control *shGFP* neurons (left panel) and *shASL* neurons (middle panel) (Interval between individual Z-stack serial images = 0.5 µm). Right panel: quantification of the fluorescence intensity of the anti-tyrosine aggregates staining of *shGFP* and *shASL* neurons supplemented with or without NO donors (*n* ≥ 14) (One-way ANOVA with Bonferroni). **C** Measurements of neuronal cell viability following the addition of the XTT reagent (*n* = 4). **D** Quantification of α-synuclein protein levels from SH-SY5Y neuronal cells (*n* = 4). Lower panel: representative western blot for α- synuclein levels. **E** ASL is located close to a PD locus variant identified by recent GWAS studies from the iPDGC Locus Browser v1.5 (59). The lead single nucleotide polymorphism (SNP) at this locus is rs76949143 (purple). Recombination rate peaks are marked in blue, and variants are colored by their r2 linkage disequilibrium values. Data represent mean ± s.e.m. (*p < 0.05, **p < 0.01, ***p < 0.001, ****p < 0.0001; *ns* not significant). **F** A summary model of our findings demonstrating that ASL loss leads to decreased TH activity, subsequently causing decreased catecholamine (CA) levels and accumulation of tyrosine which together with elevation of α- synuclein may predispose to aggregate formation

Along with a decrease in catecholamine levels, a reduction in TH enzymatic activity would be expected to increase the levels of its substrate, tyrosine. Analysis of cerebrospinal fluid (CSF) from patients with ASLD who underwent liver transplantation demonstrated higher than normal levels of tyrosine, while their plasma tyrosine levels remained within the normal ranges (Fig. 4A). These results suggest that despite the normalization of ASL function in the liver following the transplant, the tissue-autonomous loss from the central nervous system has biochemical and potentially clinically relevant consequences. Although dopamine deficiency in the SNc and aggregation of proteins in neurons are the two main hallmarks of PD, a direct connection between these findings remains indefinable (38). High levels of tyrosine have been reported to lead to neurotoxic amyloid fibrils, and tyrosine assemblies can trigger a cytotoxic effect that decreases cell viability in the SH-SY5Y neuronal cell line (39). Thus, we next evaluated the potential contribution of tyrosine accumulation to neuronal damage following ASL loss. Strikingly, knockdown of ASL in SH-SY5Y neuronal cells caused a significant deposition of tyrosine aggregates and decreased viability similar to the consequences of artificial insertion of tyrosine aggregates to neuronal cells (Fig. 4B, C). Notably, tyrosine aggregation could be reduced with NO supplementation (Fig. 4B). Reduction in catecholamine levels and specifically of norepinephrine, as we demonstrated to occur following ASL loss (8), was shown to affect the expression of α-synuclein and to promote aggregate formation (40). Thus, we next measured α-synuclein protein levels and found elevated levels in *shASL* SH-SY5Y neuronal cells compared to control cells (Fig. 4D).

Identifying tyrosine aggregates together with high α-synuclein levels suggests that ASL loss may predispose potential seeding to aggregate formation in neurons. Following the rationale of our hypothesis for possible connection between ASLD and PD, we analyzed a recent GWAS dataset of PD (41) and found that ASL is situated 0.5 Mb upstream the risk locus rs76949143 associated with PD, alluding to the potential association between genomic variants in ASL and PD (Fig. 4E). Of note, this SNP is not located in a known regulatory region for ASL.

## Discussion

ASL is the only enzyme in mammalians that can generate arginine, the substrate of NOS for the generation of NO. Individuals with ASLD suffer from neurocognitive deficits, seizures, and learning difficulties that can be out of proportion to the magnitude of hyperammonemia compared to other UCDs (4, 42–44). Additional studies in murine models have shown that neuropathology associated with ASLD can be independent of hyperammonemia (45). We and others have demonstrated that ASLD is a human model of NO deficiency and that tissue-autonomous loss of ASL can be a model to study consequences of cell-specific NO deficiency (46) (8) (47) (35) (34) (48) (7) (47). We have recently shown that ASL is expressed prominently in the noradrenergic nucleus-the locus coeruleus (LC). ASL participates in regulating catecholamine synthesis by NO *vi*a nitrosylation of TH (8, 49). Thus ASLD is a valuable model to study disease processes that involve dysregulation of NO and catecholamines like PD.

Data from humans and experimental models of PD support a role for NO in modulating the inflammation, oxidative stress, mitochondrial dysfunction, and excitotoxicity-mediated neuronal injury in PD (50). These data demonstrate that NO can play both neuroprotective and neurotoxic roles depending on the context- and cell of origin. In addition, neuronal damage is caused by both high NO levels and high levels of NO-related free radicals like nitrotyrosine, a product of tyrosine nitration mediated by peroxynitrite (51). Together, these data led us to dissect a potential role for ASL metabolism in PD in a cell-specific manner.

Here, we find that ASL is distinctly expressed in a unique subpopulation of dopaminergic neurons, the SNc–ALDH1A1^**+**^cells, which are known to have a significant vulnerability to the neuronal damage observed in PD are consequently involved in the causation of the movement-related disease manifestations. Indeed, we find that mice with loss of ASL in these dopaminergic neurons phenocopy the motor deficits observed in murine PD models. Notably, the memory deficits we observe can be affected by the parallel loss of ASL from the LC and the SNc–ALDH1A1^**+**^ cells. Interestingly, the levels of ALDH1A1, one of the biomarkers suggestive of a diagnosis of PD in humans (23), were elevated in the CSF of *Asl*^*f/f*^*; TH Cre*^+*/−*^ mice. Our data thus alludes to the potential link between ASL, TH, and catecholamine depletion to the pathogenesis of certain forms of neurodegenerative disorders involving the nigrostriatal system, such as PD.

The pathological hallmark of PD is an accumulation of protein aggregates and, specifically, of α-synuclein in the neurons of the SNc. Mechanistically, these aggregations are thought to damage neurons in the SNc and cause catecholamine levels abnormalities, mainly dopamine. To date, the cause of the accumulation and aggregation of proteins, specifically in these neurons of individuals with PD, is unknown. One potential explanation comes from the "seeding" theory, which suggests that the accumulation of amino acids may form a seed for forming larger aggregates of α-synuclein (29, 52, 53).

Inborn metabolic diseases have an inherent risk for the accumulation of different metabolites (54). Tyrosine aggregations have been described in the cornea and skin of patients with tyrosinemia type II, a congenital metabolic disease caused by deficiency of the enzyme tyrosine aminotransferase (55). Here, we describe that reducing ASL in neurons leads to reduction in TH activity and accumulation of tyrosine. This reduction in TH activity results in dual damage—a decrease in catecholamine levels and accumulation of tyrosine specifically in neurons. We further demonstrate an accumulation of tyrosine in CSF of patients with ASLD even after liver transplantation. Interestingly, patients with PD have also been shown to have high levels of tyrosine in their CSF (56, 57), and nitrotyrosine immunoreactivity has been found in the core of Lewy bodies within degenerating neurons in brains from individuals with PD (50), supporting the relevance of tyrosine accumulation in this disorder. Collectively, our data may suggest that neurons of the SNc depend on TH for dopamine synthesis and are hence particularly sensitive to tyrosine accumulation. Our findings that neuronal loss of ASL leads to elevation of α-synuclein levels further support a model in which deficiency in ASL, specifically in catecholamine synthesis neurons, causes a reduction in TH levels that result in accumulation of tyrosine that can potentially lead to aggregation in TH neurons (Fig. 4F).

Rare disorders as inborn errors of metabolism, offer us a unique prism to understand the contribution of mutations in single enzymes to the pathogenesis of complex diseases (58). Here, we illustrate this point by dissecting ASL's role in the SNc and shed light on the potential metabolic association between ASL and SNc-related neurodegenerative disorders such as PD. Importantly, our work suggests translational implications for further exploring the possible use of NO supplementation in dopaminergic-related pathologies.

## Methods

### Human studies

The Urea Cycle Disorders Consortium (UCDC) of the National Institutes of Health Rare Diseases Clinical Research Network (RDCRN) consists of 16 clinical sites across the United States, Canada, and Europe. Clinical data presented in this report were collected in a standardized format according to a manual of operations as part of the Longitudinal Study of Urea Cycle Disorders (NCT00237315), an ongoing natural history study conducted by the consortium since 2006. This study was approved by the Institutional Review Boards (IRB) of all clinical sites of the UCDC between 2006 and 2015. Since 2015, the IRB at Children's National Medical Center has served as the consortium's central IRB. Informed consent was obtained from all participants or their parents or legal guardians. For the Longitudinal Study of Urea Cycle Disorders, clinical, laboratory, and neuropsychological data are collected. For the analyses presented here, the following data were collected from individuals with ASLD and ASS1D: gender, age, presence or absence of at least one hyperammonemia episode (defined as plasma ammonia levels greater than 100 µmol/L), presence or absence of tremors.

### Human CSF samples

CSF and plasma samples were obtained as part of the standard liver transplant protocol at Rome's Bambino Gesù Childrens Hospital. The studies were performed under the Declaration of Helsinki and approved by the Ethical Committee (2119_OPBG_2020). The age of the patients ranges between 2.5 and 12.0 years.

### GWAS analysis

Method: Parkinson's Disease GWAS Locus Browser. The iPDGC Locus Browser v1.3.1 (https://pdgenetics.shinyapps.io/GWASBrowser/) combines data from multiple databases and recent large-scale genome-wide association studies (GWAS) for Parkinson's disease (PD)(41)(59). 92 genome-wide significant PD GWAS variant loci are presented with the genes most associated with the variant of interest 1 Megabase (Mb) up and downstream based on self-ranked criteria and the hg19 reference genome (60).

### Animal studies

All animal procedures were approved by the Institutional Animal Care and Use Committee (applications number: 07201118-1) and were performed in strict adherence to Weizmann Institute Animal Care and Use guidelines. C57BL/6JOlaHsd mice were purchased from ENVIGO RMS (ISRAEL). The B6.Cg-Tg(Th-cre)1Tmd/J were kindly given to us by Dr. Ofer Yizhar (61). Mice were monitored daily by Weizmann Institute staff and veterinary personnel for health and activity. Mice were given ad libitum access to water and standard mouse chow with 12-h light/dark cycles. Littermates of age and gender-matched mice were randomly assigned to experimental groups.

### Cell cultures

SH-SY5Y human neuroblastoma cells (American Type Culture Collection, ATCC, Manassas, VA, USA) were grown in DMEM (Dulbecco's modified Eagle's medium) supplemented with 10% heat-inactivated FBS, 100 units/mL streptomycin, and 100 μg/mL penicillin at 37 °C in a humidified 5% CO_2_ atmosphere. All cells were tested routinely for mycoplasma using a Mycoplasma EZ-PCR test kit (20–700-20, Biological Industries).

### Virus infection

HEK293T cells were used for packaging the lentivirus. HEK293T cells in the logarithmic growth phase were seeded into a 10 cm plate. Once cell confluence reached 80%, viral packaging mix (Renium K4975-00) and 1 µg of either *shGFP* or *shASL* were co-transfected into HEK293T cells with the aid of lipofectamine 2000. 48 h after transfection, the supernatant of HEK293T cells was collected and centrifuged (1000 rpm) at 4 °C for 10 min to remove cell debris. SH-SY5Y cells were seeded in 6-well plates (200,000 cells/well) and grown to reach approximately 80% confluency. 0.5 ml of the supernatant was added to the cell culture medium without Penicillin–Streptomycin and incubated with the cells for 12 h. On the next day, the medium was replaced by a fresh medium. The cells were then transferred to a 10 cm plate, and Puromycin (4 μg/mL) was added for 4 days.

### Cell viability experiments

Cells were incubated in a medium without serum for 24 h (100 μl). After incubation, cell viability was evaluated using the XTT cell proliferation assay kit (Biological Industries) according to the manufacturer's instructions. Briefly, 100 μl of the activation reagent was added to 5 ml of the XTT reagent, followed by 50 μl of activated XTT solution to each well. After 3 h of incubation at 37 °C, color intensity was measured using an enzyme-linked immunosorbent assay (ELISA) microplate reader at 450 and 630 nm.

### Mice perfusion

Following euthanasia, mice were rapidly perfused with cold 4% paraformaldehyde (PFA) in PBS. Mice brains were quickly removed, immersed in freshly 4% PFA for 24 h, and then transmitted to 1% PFA for 24 h. For *in-situ* hybridization, mouse brains were placed in 1 × PBS with 30% sucrose for another 24 h and then frozen and sectioned in the coronal plane using a microtome. For immunostaining, mouse brains were embedded in paraffin.

### NO treatment

For in vitro assays, 100 μM S-Nitroso-N-acetyl-DL-penicillamine (SNAP) (CayMan chemicals 82,250) was added to the medium 24 h before cell collection. For in vivo rescue experiments, mice were treated with NaNO_2_ 100 mg/kg/d in drinking water, renewed every 3 days (Sigma-Aldrich, St. Louis, MO, catalog number S2252).

### Catecholamine analysis

#### Extraction

The extraction procedure was performed at 4 °C. Pre-weighted samples in 1.5-mL test tubes were spin shortly (21,000 g, 15 s) to place them at the bottom. 50 μL of 4% perchloric acid containing IS mix (NorLeu 2 μM; Arg-13C6 12 μM; NEN-D6 20 ng/ml; DA-D4 40 ng/ml; ST-D4 400 ng/ml) was added, and the mixture was homogenized using a handheld grinder (Agros), followed by agitation in a shaker (1200 rpm, 30 min, ThermoMixer C, Eppendorf) and centrifuged (20,000 g, 10 min). The collected supernatants were used for further analysis.

#### Derivatization

Derivatization procedure was performed using AQC reagent synthesized as described (62). Briefly, a 10-μL aliquot of the sample or standard solution (with the internal standards added) and 70 μL of 0.15 M sodium borate solution, pH 8.8 were derivatized with 20 μL of AQC in acetonitrile (2.7 mg/mL) by heating at 55 °C for 10 min. The reaction mixtures were cooled and placed in nanofilter vials (Thomson, 0.2 μm PES) for LC–MS.

#### LC–MS/MS analysis

The LC–MS/MS instrument consisted of Acquity I-class UPLC system (Waters) and Xevo TQ-S triple quadrupole mass spectrometer (Waters) equipped with an electrospray ion source and operated in positive ion mode was used for analysis. MassLynx and TargetLynx software (v.4.1, Waters) was applied to acquire and analyze data. Chromatographic separation was done on a 150 × 2.1-mm i.d. 1.8-µm UPLC HSS T3 column equipped with 50 × 2.1-mm i.d., 1.8-µm UPLC HSS T3 pre-column (both Waters Acquity) with 0.1% formic acid as mobile phase A and 0.1% formic acid in acetonitrile as B at a flow rate of 0.6 ml/min and column temperature 45 °C. A gradient was as follows: for 0.5 min, the column was held at 4%B, then linear increase to 10%B in 2 min, then to 28%B in 2.5 min, and to 95%B in 0.1 min. Just after back to 0%B during 1.1 min, and equilibration at 4%B for 1.3 min. Samples kept at ambient temperature (23 °C) were automatically injected in a volume of 1 μl. For mass spectrometry, argon was used as the collision gas with flow of 0.10 ml/min. The capillary voltage was set to 3.00 kV, cone voltage 25 V, source offset 30 V, source temperature 150 °C, desolvation temperature 650 °C, desolvation gas flow 800 L/hr, cone gas flow 150 L/hr. Analytes were detected using corresponding selected reaction monitoring (SRM) and retention times, as shown in the table. The concentrations based on standard curves were calculated using TargetLynx (Waters).

### Laser microdissection (LMD)

Brains were removed rapidly, briefly washed in cold PBS, embedded, and frozen in OCT on dry ice without fixation. 8 μm thick sections were cut from the frozen block, mounted on polyethylene membrane-coated glass slides (Zeiss, A4151909081000), air-dried for 1 min at room temperature, and put in -80 °C. On the day of the experiment, slides were thawed for 5 min at room temperature followed by fixation in 70% ethanol (30 s), incubation in DEPC water (1 min), stained with TH antibody (ab209921, 10 min), washed vigorously with PBS (30 s) and air-dried for 3 min before microdissection. The cutting was performed with the following parameters: PALM 20X lens, cut energy 45 (1–100), cut focus 65 (1–100). Tissue fragments were catapulted and collected in 0.2 ml adhesive cap tubes (Zeiss, A4151909181000) with these settings: LPC energy 50 (1–100), LPC focus 63 (1–100). After the collection session, the capturing success was visually confirmed by focusing the PALM on the targeted adhesive cap.

### Behavioral studies

#### CatWalk

This system was used for the quantitative assessment of footfalls and gait. The apparatus consists of an enclosed walkway that a mouse walks on; the "Illuminated Footprints" technology allows a high-speed video camera (positioned underneath the walkway) to capture the footprints (Noldus, Wageningen, Netherlands). These images are processed based on the dimensions, position, and dynamics of each footfall to produce quantitative analyses of footfalls and gait: each mouse went through a test session that was comprised of five "runs" (the mouse walks the full length of a 50 cm runway) that comply with minimal speed variation requirements (less than 40%).

#### Morris water maze

We studied possible alterations of spatial memory in the Morris water maze. The water maze consisted of a circular tank (120 cm diameter) with a removable escape platform centered in one of the four maze quadrants. In the testing room, only distal visual-spatial cues for locating the hidden platform were available. During testing, the tank is filled with 24 ˚C water clouded with milk powder. The mice were subjected to 4 trials per day with an inter-trial interval of 10 min, for 7 consecutive days. In each trial, the mice were required to find a platform located in one of the four quadrant submerged 1 cm below the water surface. The escape latency in each trial was recorded up to 90 s. Each mouse was allowed to remain on the platform for 15 s and was then removed from the maze. If the mouse did not escape in the allocated time, it was manually placed on the platform for 15 s. Memory was assessed 24 h after the last trial. The escape platform was removed and mice were allowed to search for it for 1 min, and the time spent swimming in the different quadrants of the pool was monitored using an automated tracking system (Noldus, Wageningen, Netherlands).

#### Fear conditioning

The fear-conditioning paradigm was used to study possible alteration of hippocampal or amygdala-dependent forms of memories. A computer-controlled fear-conditioning system (Noldus, Wageningen, Netherlands) monitors the procedure while measuring freezing behavior (i.e., lack of movement except respiration). The test is performed within 3 days as previously described (63): 1) Habituation: on the first day, mice are habituated for 5 min to the fear conditioning chamber, a clear Plexiglas cage (21 cm × 20 cm × 36 cm) with a stainless steel floor grid within a constantly illuminated (250 lx) fear-conditioning housing. 2) Conditioning: conditioning takes place on day 2 in one 5-min training session. Mice initially explore the context for 2 min. After that, two pairings of a co-terminating tone [conditioned stimulus (CS): 30 s, 3,000 Hz, pulsed 10 Hz, 80 dB (A)] and shock [unconditioned stimulus (US): 0.7 mA, 2 s, constant current) with a fixed ITI of 60 s. The US is delivered through the metal grid floor. Mice are removed from this chamber 1 min after the last CS-US pairing. The chamber is cleaned with 10% ethanol before each session. The ventilating fan of the conditioning box housing provides a constant auditory background noise [white noise, 62 dB(A)]. 3) Testing: Context-dependent memory is tested 24 h after the conditioning by re-exposure to the conditioning box for 5 min without any stimuli. The Cue dependent memory is tested 1 h after the Context test by exposure to the conditioned [conditioned stimulus (CS): 30 s, 3,000 Hz, pulsed 10 Hz, 80 dB (A)] in different environmental conditions (black Plexiglas box, black floor instead of metal grid, no illumination, no ventilation noise, cleaning solution: acetic acid 10% instead of alcohol 10%).

#### Brain tissue biopsies collection

Immediately after decapitation, mouse brains were removed and placed in a 1.0 mm coronal slice intervals brain matrix (BSMAS001-1 Civic Instruments). The brains were sliced using standard razor blades into 2-mm slices that were frozen immediately on dry ice. The areas of interest were punched using a micro dissecting 16G needle according to the anatomical references of The Mouse Brain in Stereotaxic Coordinates (axinos F, Franklin KBJ (2001)). The brains were stored at − 80 °C immediately for later use.

### Immunostaining

In-vivo: four-micrometer paraffin-embedded tissue sections were deparaffinized by xylene and rehydrated through a gradient of ethanol. Sections were exposed to acetone for 7 min at − 20 °C, and antigens were retrieved in citric acid in a microwave oven at full intensity for 3 min until a boiling point was reached, and then at 20% intensity for 10 min. Blocking nonspecific binding was done with 20% normal horse serum and 0.2–0.5% triton for 90 min in a humidity chamber. Sections were incubated with the primary antibodies: ASL (1:100, Abcam, ab97370); TH (1:500, millipore AB1542). All antibodies were diluted in PBS containing 2% normal horse serum and 0.2% Triton. Sections were incubated overnight at RT followed by 48 h at 4 °C. Sections were washed three times in PBS and incubated with the biotinylated anti-rabbit antibody for 90 min in a humidity chamber, washed, and incubated with streptavidin Cy2 and Cy3 anti-goat antibodies (all from Jackson ImmunoResearch) for 40 min. Sections were counterstained by Hoechst (Molecular Probes). Stained sections were examined and photographed with a fluorescence microscope (Eclipse Ni-U; Nikon) equipped with Plan Fluor objectives (20x;40x) connected to a color camera (DS-Ri1, Nikon) microscope. Excitation/emission wavelengths were 412/450 nm for DAPI and 548/561 nm for Cy3

In-vitro: cells were grown to 70% confluence on poly-L-lysine coated coverslips in 24-well plates. The cells were then rinsed with PBS and fixed in 4% PFA for 15 min at room temperature. The cells were washed twice with ice-cold PBS and treated with 0.25% Triton X-100 for 10 min at room temperature to allow cellular permeabilization. After thoroughly washing the cells, blocking was performed using 1% BSA for 30 min at room temperature. Then, the cells were stained using a rabbit polyclonal anti-tyrosine antibody diluted 1:200 in blocking solution for overnight incubation at 4 ^◦^C. The slides were washed three times with PBS, and an anti-rabbit Cy3-conjugated secondary antibody diluted 1:200 in blocking solution was added for 30 min at room temperature in the dark. Finally, cells were washed three times, and the coverslips were mounted using 15 µL Vectashield Antifade Mounting Medium with DAPI. Imaging was performed using SP8 inverted confocal microscopy. Excitation/emission wavelengths were 412/450 nm for DAPI and 548/561 nm for Cy3. The fluorescence intensity was quantified using ImageJ software.

### Western blotting

Cells were lysed in RIPA and 1:100 protease inhibitor (Sigma-Aldrich). After centrifugation, the supernatant was collected, and protein content was evaluated by the BCA protein assay kit (Thermo Fisher 23225). 80 µg of each sample under reducing conditions were loaded into each lane and separated by electrophoresis on a 10% SDS polyacrylamide gel. Following electrophoresis, proteins were transferred to Immobilon transfer membranes (Tamar). Non-specific binding was blocked by incubation with 5% milk in TBST (10 mM Tris–HCl (pH 8.0), 150 mM NaCl, 0.1% Tween 20) for 1 h at 25 °C. Membranes were subsequently incubated with antibodies against ASL (1:500, ab97370, Abcam), p97 (1:10,000, PA5-22257, Thermo Scientific), GAPDH (1:1,0000, ab128915, Abcam), TH (1:500, CST-2792S, cell signaling) α-synuclein. Antibody was detected using peroxidase-conjugated AffiniPure goat anti-rabbit IgG or goat anti-mouse IgG (Jackson ImmunoResearch) and enhanced using chemiluminescence western blotting detection reagents (Pierce™ ECL Western Blotting Substrate, Thermo Fisher). Gels were quantified by Gel Doc XR + (BioRad) and analyzed by ImageLab 6.0 software (BioRad). The band area was calculated by the band's intensity divided by the value obtained from the loading control.

### RNA extraction and complementary DNA (cDNA) synthesis

RNA was extracted from cells using RNeasy Mini Kit (74104, QIAGEN). cDNA was synthesized from 1 μg RNA using qScript cDNA Synthesis Kit (Quanta). Quantitative PCR was performed using SYBR green PCR master mix (Thermo Fisher scientific 4385612) or TaqMan Real-Time PCR Master Mix (Thermo Fisher scientific 4444557).

### Proteomic analysis for CSF collection

#### Sample preparation

All chemicals were ordered from Sigma unless otherwise noted. 10–20 µl of CSF were mixed with 8 M urea. Proteins were reduced with 5 mM dithiothreitol for 1 h at room temperature and alkylated with 10 mM iodoacetamide in the dark for 45 min at room temperature. Samples were diluted to 2 M urea with 50 mM ammonium bicarbonate. Proteins were then subjected to digestion with trypsin (Promega; Madison, WI, USA) overnight at 37 °C at 50:1 protein: trypsin ratio, followed by second trypsin digestion for 4 h. The digestions were stopped by the addition of trifluoroacetic acid (1% final concentration). Following digestion, peptides were desalted using Oasis HLB, μElution format (Waters, Milford, MA, USA). The samples were vacuum dried and stored at −80 °C until further analysis.

#### Liquid chromatography

ULC/MS grade solvents were used for all chromatographic steps. Each sample was loaded using split-less nano-ultra performance liquid chromatography (10 kpsi nanoAcquity; Waters, Milford, MA, USA). The mobile phase was: A) H_2_O + 0.1% formic acid and B) acetonitrile + 0.1% formic acid. Desalting of the samples was performed online using a reversed-phase Symmetry C18 trapping column (180 µm internal diameter, 20 mm length, 5 µm particle size; Waters). The peptides were then separated using a T3 HSS nano-column (75 µm internal diameter, 250 mm length, 1.8 µm particle size; Waters) at 0.35 µL/min. Peptides were eluted from the column into the mass spectrometer using the following gradient: 4% to 27%B in 105 min, 27% to 90%B in 5 min, maintained at 90% for 5 min and then back to initial conditions.

#### Mass spectrometry

The nanoUPLC was coupled online through a nanoESI emitter (10 μm tips; New Objective; Woburn, MA, USA) to a quadrupole orbitrap mass spectrometer (HFX, Thermo Scientific) using a FlexIon nanospray apparatus (Proxeon). Data were acquired in data-dependent acquisition (DDA) mode, using a Top10 method. MS1 resolution was set to 120,000 (at 400 *m*/*z*), a mass range of 375–1650 *m*/*z*, AGC of 1e6, and maximum injection time was set to 60 ms. MS2 resolution was set to 15,000, quadrupole isolation 1.7 *m*/*z*, AGC of 1e5, dynamic exclusion of 30 s, and maximum injection time of 60 ms. A preferential inclusion list was specified for the higher priority of MS/MS triggering. The list of peptides is provided as a Supplementary File.

#### Data processing

Raw data were processed with MaxQuant v1.6.6.0 using the default parameters except the following: LFQ min. Ratio count = 1, separate LFQ in parameter groups (young mice vs. old mice), and match between runs was enabled. The data were searched with the Andromeda search engine against the murine proteome database (November 2019 version) appended with common lab protein contaminants and the following modifications: carbamidomethylation of C as a fixed modification and oxidation of M and protein N-terminal acetylation as variable ones. The LFQ (Label-Free Quantification) intensities were calculated and used for further calculations using Perseus v1.6.2.3. Decoy hits were filtered out, as well as proteins that were identified based on a modified peptide only. The LFQ intensities were log2 transformed, and only proteins with at least 4 valid values in at least one experimental group were kept. The remaining missing values were imputed. Statistical analysis was done using a Student's t-test.

### Statistical analysis

Unless indicated otherwise, values are expressed as mean ± SEM. As appropriate, statistical analysis was performed using repeated-measures two-way ANOVA with Bonferroni post hoc *t* tests or Student's *t* tests. Statistical details of individual experiments, such as exact values of , can be found in figures and legends. The sample size was chosen in advance based on the common practice of the described experiment and is mentioned for each experiment. Each experiment was conducted with biological and technical replicates and repeated at least three times. Statistical tests were done using Prism software (GraphPad Software). *P* < 0.05 was considered significant in all analyses (* denotes *P* < 0.05, **P < 0.005, ***P < 0.0005, ****P < 0.0001).

## Supplementary Information

Below is the link to the electronic supplementary material.

**Supplementary figure 1: ASL KO in ALDH1A1**^**+**^**neurons in the SNc.** (**A**) ASL expression in the SNc of wild-type mice (top left) and co-localized with TH (top right). ASL and TH are deficient specifically in the SNcM (indicated by dashed line) of *Asl*^*f/f*^*; TH Cre*^*+/−*^ mice (lower panel). (Scale bar=250 µm). (**B**) A representative fresh-frozen brain section of the SNc and VTA was stained with TH antibody before (left panel) and after laser microdissection (right panel). (**C**) Quantification of *Asl* mRNA isolated by laser microdissection from the LC of *Asl*^*f/f*^*; TH Cre*^*+/−*^ and from *Asl*^*f/f*^ control mice as measured by RT-PCR with specific TaqMan probes (n=7 mice in each group). (TIF 24724 KB)

**Supplementary figure 2: ASL KO in catecholamine neurons results in abnormal TH levels in the SNc and abnormal ALDH1A1 levels in the CSF.** (**A**) Representative images of TH expression in ALDH1A1^**+**^ neurons in the SNcM. Dashed lines differentiate the SNc and VTA subregions. (**B**) ALDH1A1protein levels in the CSF of adult *Asl*^*f/f*^*; TH Cre*^*+/−*^ and *Asl*^*f/f*^ control mice (n=5 mice in each group). SNcM-SNc medial, SNcD-SNc dorsal, PBP-parabrachial pigmented nucleus. (TIF 19955 KB)

**Supplementary figure 3: Adult Asl**^***f/f***^***; TH Cre***^***+/−***^** mice demonstrate long memory impairments in the Morris Water Maze test. **(**A**) Spatial memory was evaluated two days following the last training session. Adult *Asl*^*f/f*^ control group spent significantly longer times in the target quarter than adult *Asl*^*f/f*^*; TH Cre*^*+/−*^ mice. (**B**) For 7 consecutive learning days, adult mice did not show any significant differences in the length of time spent finding the platform (n$\ge$ 8). Data represent mean ± s.e.m. (*p < 0.05). (TIF 5319 KB)
