# Supplementary figures and images for: ASL expression in ALDH1A1+ neurons in the substantia nigra metabolically contributes to neurodegenerative phenotype
Source: Hum Genet. 2021 Aug 21;140(10):1471–85. doi: 10.1007/s00439-021-02345-5 (PMC8460544; doi:10.1007/s00439-021-02345-5)

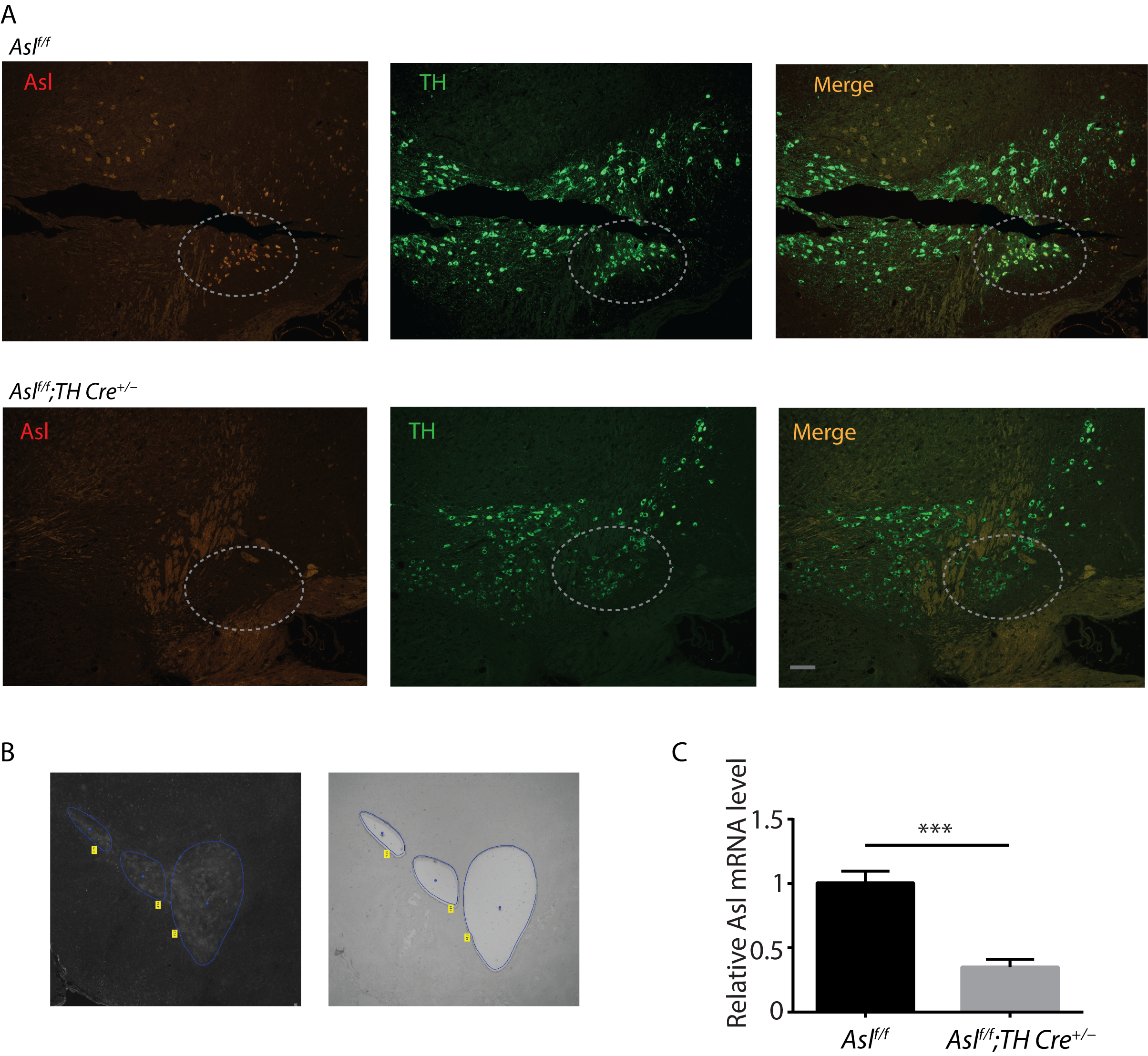

Supplement: Supplementary file 1 — Supplementary figure 1: ASL KO in ALDH1A1+neurons in the SNc. (A) ASL expression in the SNc of wild-type mice (top left) and co-localized with TH (top right). ASL and TH are deficient specifically in the SNcM (indicated by dashed line) of Aslf/f; TH Cre+/− mice (lower panel). (Scale bar=250 µm). (B) A representative fresh-frozen brain section of the SNc and VTA was stained with TH antibody before (left panel) and after laser microdissection (right panel). (C) Quantification of Asl mRNA isolated by laser microdissection from the LC of Aslf/f; TH Cre+/− and from Aslf/f control mice as measured by RT-PCR with specific TaqMan probes (n=7 mice in each group). (TIF 24724 KB) [file 439_2021_2345_MOESM1_ESM.tif]

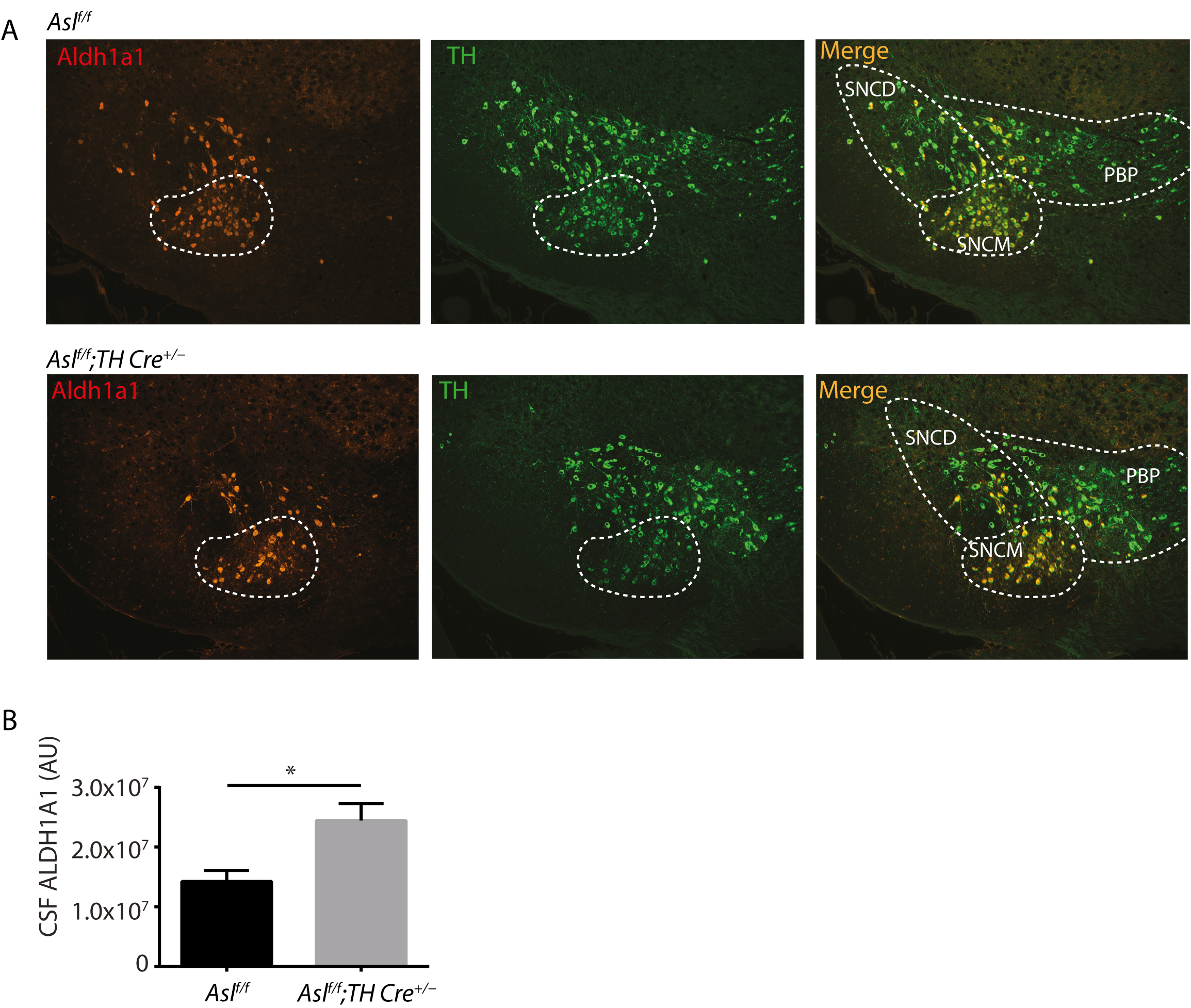

Supplement: Supplementary file 2 — Supplementary figure 2: ASL KO in catecholamine neurons results in abnormal TH levels in the SNc and abnormal ALDH1A1 levels in the CSF. (A) Representative images of TH expression in ALDH1A1+ neurons in the SNcM. Dashed lines differentiate the SNc and VTA subregions. (B) ALDH1A1protein levels in the CSF of adult Aslf/f; TH Cre+/− and Aslf/f control mice (n=5 mice in each group). SNcM-SNc medial, SNcD-SNc dorsal, PBP-parabrachial pigmented nucleus. (TIF 19955 KB) [file 439_2021_2345_MOESM2_ESM.tif]

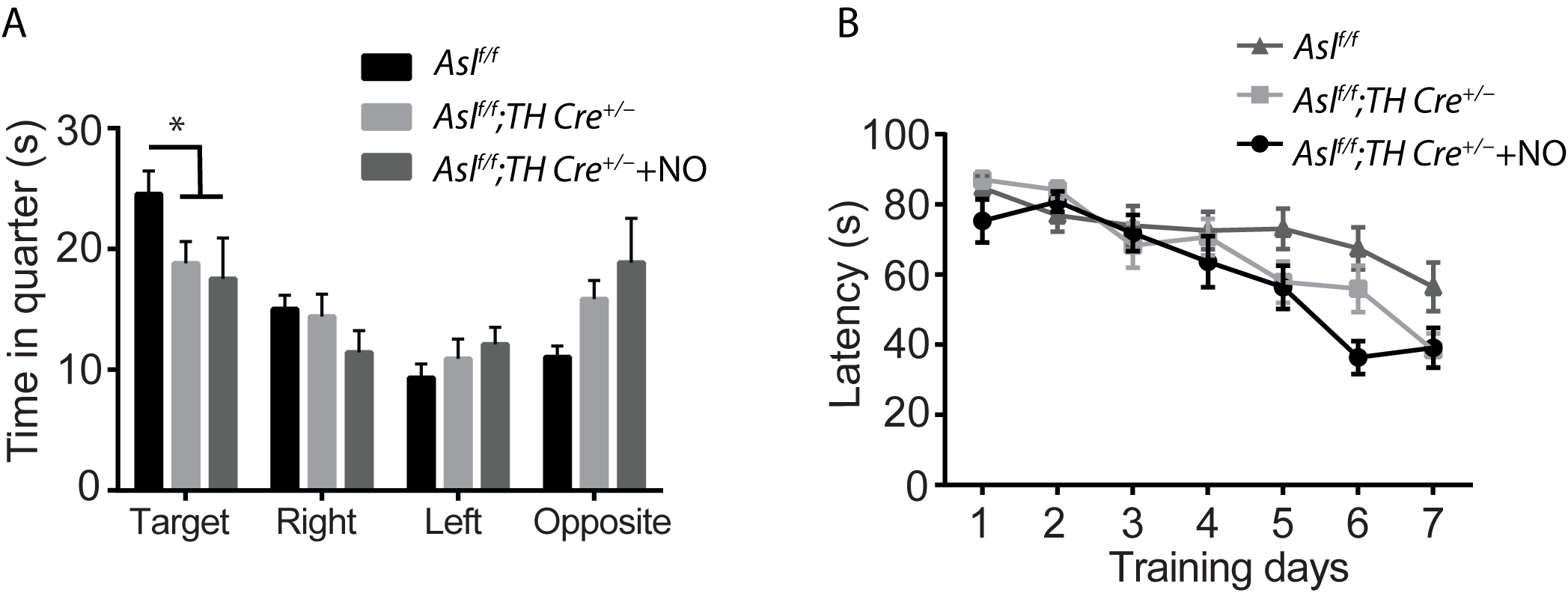

Supplement: Supplementary file 3 — Supplementary figure 3: Adult Aslf/f; TH Cre+/− mice demonstrate long memory impairments in the Morris Water Maze test. (A) Spatial memory was evaluated two days following the last training session. Adult Aslf/f control group spent significantly longer times in the target quarter than adult Aslf/f; TH Cre+/− mice. (B) For 7 consecutive learning days, adult mice did not show any significant differences in the length of time spent finding the platform (n$\ge$≥ 8). Data represent mean ± s.e.m. (*p < 0.05). (TIF 5319 KB) [file 439_2021_2345_MOESM3_ESM.tif]
